# Comparing general practice in Australia and in England: an Australian perspective and the RCGP International and Overseas Network

**DOI:** 10.3399/bjgpopen18X101577

**Published:** 2018-06-13

**Authors:** Jagdeesh Singh Dhaliwal

**Affiliations:** 1 Founder Member, International and Overseas Network, Royal College of General Practitioners, London, UK; 2 Member, Editorial Board, Australian Journal of General Practice, Melbourne, Australia; 3 Medical Director, My Home GP, Melbourne, Australia; 4 Clinical Director, Aged Care GP, Melbourne, Australia; 5 Clinical Council Member, South Eastern Melbourne Primary Health Network, Melbourne, Australia; 6 Member, Medical Education Advisory Board, Reed Medical Education, Melbourne, Australia

**Keywords:** international and overseas network, general practice, United Kingdom, Australia

## Introduction

Participation as a founder member of the International and Overseas Network (ION) of the Royal College of General Practitioners (RCGP) presented me with the fortuitous opportunity and challenge to pause, to reflect, and to take stock of my experience of life as a GP in Australia and as a GP in England. This article compares and contrasts the primary healthcare systems in both countries from the vista of the individual, practising GP working in those systems, and considers the approaches and developments that each system might learn from the other.

Following 20 years as a coalface GP, who has also held various NHS management and healthcare leadership teaching positions, I departed the UK with my family in 2016 to follow my wife as she stepped forward to accept an exciting new corporate job opportunity. Contented as a GP in England, emigrating — for me, as an individual — was categorically about ‘moving towards something new’ rather than an expression of dissatisfaction with my career or life in England.^1^

Frequent immersion back in the UK — five visits in 18 months, interspersed between growth and consolidation of experience in Australia — has allowed for an evolving and reflexive^2^ overview^3^ of the two health systems from the standpoint of the individual GP.

My experience in England was as a GP practising in a relatively affluent socioeconomic locale. My Australian experience is in a similar socioeconomic urban setting. As such, my reflections are drawn from comparing and contrasting the healthcare systems’ impact in these specific contexts. GPs practising in rural and remote settings, or those working with indigenous communities, will have different experiences of the UK and Australia. Further, a variety of healthcare reforms are underway in Australia which will alter this experience, just as UK general practice will change and develop with new policies and initiatives.

What then, from my personal case-study experiences, would I suggest Australia learn from the UK and the UK learn from Australia?

The global financial crisis (GFC) hit health services hard across Europe,^4^ whereas Australia — ‘The Lucky Country’ — remained relatively unscathed.^5^

The GFC, like any environmental shock, represented a seismic threat and also an opportunity to all sections of society and the economy, including health care.^4^

The impact as a British GP was austerity, severe funding cuts, a recruitment crisis, and the ‘scorched earth event’ that created ecological space to offer promising disruptive technologies^6^ the niche to take root and thrive. The period from 2010–2016 saw the growing, day-by-day impact on me, as a GP and a practice-owner, of innovations such as the NHS e-referrals service,^7^ and the electronic prescriptions service.^8^ NHS policy placed digital technology promotion centre-stage, and cultural changes encouraged full patient access to their digital records.^9,10^

Notwithstanding the failures, the fudges, and the frustrations of technology implementation, and of staff adoption and adaptation, the net impact as a practitioner in the UK is a system where the infrastructure — the ‘train tracks’ — for digital transformation exist. This allows the individual GP to encourage patients and their families to access their full medical records, including clinical progress notes, results, and hospital letters; it enables patients to view information online before and after consultations using ‘safe search’ options facilitated by their GP practice; and it allows GPs to make optimal use of m-health and app technologies to interact with patients in between their consultations.^11,12^

Australia has the My Health Record online health summary initiative,^13^ and many examples of available homegrown and overseas-produced apps,^14^ but the infrastructure that might offer patients the choice to undertake an online search comparing public health providers, to access their full medical records as opposed to a summary, or to request and receive prescriptions via a completely digital prescribing service are not currently in place.

It’s noticeable how the primary care system in Australia gives less prominence to nurses in urban settings: advanced nurse practitioner consultations and chronic disease management clinics have burgeoned in the UK, following the pressures on the NHS, whereas in Australia, doctors are frequently involved in work that’s typically nurse-led in the UK, such as assessing wounds, recommending dressings, and managing travel immunisations. The distinct funding models — item-of-service in Australia, and capitation in the UK — are the likely root causes for these differences and drivers for efficiency.^15^

## What might the UK learn from Australia?

Patients and practitioners in Australia are aware, and are reminded every day, of the cost of each healthcare interaction: practitioners are required to bill the patient via Medicare,^16 ^our national health system, for each service provided to a patient. Each consultation attracts a fee, and Medicare reimburses patients a fixed dollar amount for GP consultations. Patients are used to paying for many aspects of health care and there are no completely free prescriptions for any resident, regardless of age, but an annual safety net guarantees a maximum amount each year each Australian will pay before the state steps in to shoulder the burden. A safety net also exists for our Australian Aboriginal and Torres Strait Islander patients. The net impact, for me, feels like a more open and transparent discussion about money, and the cost of doing business in health care in Australia. This contrasts with what often feels like the perennial pressure to conjure yet another efficiency rabbit out of the shrinking NHS budget hat. Patients in Australia can arguably exercise more agency in exploring their public and private healthcare options, and innovators seem to be more able to test out new approaches thanks to direct funding by satisfied consumers, rather than wading through the interminable NHS procurement process which will inevitably favour larger incumbents rather than promising new start-ups. GPs also have the flexibility to request additional patient co-payments for their services, and patients are free to shop around for services from other providers. This can mean, for example, charging a premium for peak-hour evening and weekend services, while requiring no additional payment for less popular daytime services. The net result is a healthcare conversation about money which — in the urban, affluent environment in which I practice in Australia — feels refreshing and empowering to patients, compared to our culture of ‘free health care is a right that I am owed’ in the UK. However, this is not the case, I understand, for GPs who work with indigenous communities and disadvantaged populations, where service and prescription fees certainly do act as a significant barrier to healthcare equity.^17^

## What would I suggest to my GP colleagues in Australia?

Australia ducked the burning platform of the GFC, but risks being slowly boiled by the economic pressures faced by all other OECD (Organisation for Economic Co-operation and Development) healthcare systems.^18^ As individual GPs, and as members of larger state and national organisations, we need to anticipate this future, and embrace opportunities now to trial and test promising disruptive digital technologies and novel teamworking practices that maximise rather than retrench skill-mix.

## What would I suggest to GP colleagues in the UK?

Make healthcare costs more evident and obvious to the British public. A bill for each GP consultation, for each medicine based on its actual cost, for each allied health professional attendance, for each hospital stay with a subsequent line item demonstrating the 100% NHS rebate. Explore the possibility of opening up patient choice, to allow co-payment for non-standard GP services; practice-based dermatology or fee-paying telephone consultations at the weekend with a named GP partner, for example. This might encourage a shift in attitudes towards the place of money and costs in health care, and our patients may arguably become, in transactional^19^ terms, more empowered and equal partners rather than dependent recipients. The potential value of these ideas would have been lost to me, and may well have seemed disturbing, when all I knew was life as a GP in the NHS.

Australia and UK have the second and the first rankings, respectively, in a review of healthcare systems^17^ conducted by the Commonwealth Fund. This report highlighted strengths and areas for improvement that each healthcare system could learn from others. Indeed, it is likely that each healthcare system around the world has something to teach and something to learn. Flying visits by the great and the good are one thing, but true organisational learning is probably most helped by immersion.^20^ The RCGP, the Royal Australian College of General Practitioners, and their sister colleges around the world could best help their members by examining ways to streamline mutual recognition, and to lobby to facilitate the easy movement of GPs between countries to allow for sabbaticals and exchanges: we, as practitioners, will gain from the rich contrast in experience; our practices will gain from new and complementary skillsets; our profession will gain from a reimagining of what and how health care can function; and the sum total of this activity could help to invigorate standards and experiences of quality health care for our patients.
